# Limbal stromal cells derived from porcine tissue demonstrate mesenchymal characteristics ***in vitro***

**DOI:** 10.1038/s41598-017-06898-2

**Published:** 2017-07-25

**Authors:** Julia Fernández-Pérez, Marcus Binner, Carsten Werner, Laura J. Bray

**Affiliations:** 1 Leibniz Institute of Polymer Research Dresden, Max Bergmann Center of Biomaterials Dresden, Center of Regenerative Therapies, Hohe Straße 6, Dresden, Saxony 01069 Germany; 2 Dresden University of Technology, Dresden, Saxony 01069 Germany; 3 Queensland University of Technology (QUT), Queensland 4059 Kelvin Grove, Australia; 4 Present Address: Trinity Centre for Bioengineering, Trinity Biomedical Science Institute, Trinity College Dublin 2, Dublin, Ireland

## Abstract

Limbal stromal cells (LSCs) from the human ocular surface display mesenchymal stromal cell characteristics *in vitro*. In this study, we isolated cells from the porcine limbal stroma (pLSCs), characterised them, and evaluated their ability to support angiogenesis and the culture of porcine limbal epithelial stem cells (pLESCs). The isolated cells adhered to plastic and grew in monolayers *in vitro* using serum-supplemented or serum-free medium. The pLSCs demonstrated expression of CD29, and cross-reactivity with anti-human CD45, CD90, CD105, CD146, and HLA-ABC. However, expression of CD105, CD146 and HLA-ABC reduced when cultured in serum-free medium. PLSCs did not undergo adipogenic or osteogenic differentiation, but differentiated towards the chondrogenic lineage. Isolated cells were also co-cultured with human umbilical vein endothelial cells (HUVECs) in star-shaped Poly(ethylene glycol) (starPEG)-heparin hydrogels to assess their pericyte capacity which supported angiogenesis networks of HUVECs. PLSCs supported the three dimensional HUVEC network for 7 days. The isolated cells were further growth-arrested and evaluated as feeder cells for pLESC expansion on silk fibroin membranes, as a potential carrier material for transplantation. PLSCs supported the growth of pLESCs comparably to murine 3T3 cells. In conclusion, although pLSCs were not completely comparable to their human counterpart, they display several mesenchymal-like characteristics *in vitro*.

## Introduction

The limbus serves as the stem cell niche for epithelial progenitor cells, which are responsible for the repair of damaged corneal epithelium. More precisely, epithelial stem cells are located in the limbal epithelial crypts^1^, which provide a unique microenvironment for the maintenance of the stem cell population. The functional and/or anatomical loss of limbal epithelial stem cells (LESCs) results in Limbal Stem Cell Deficiency (LSCD), and leads to inflammation, scarring, neovascularisation and in some cases blindness. This loss can be due to chemical or thermal burns, radiation, genetic or autoimmune disorders, contact lens use, infection or drug use^2^.

The fate of LESCs within their niche is influenced by the stromal microenvironment. In the limbal stroma there is a vascular plexus, containing smooth muscle cells, vascular endothelial cells and pericytes, which surround and support the endothelial cells, enhancing angiogenesis and protecting them from apoptosis. Fibroblasts and dendritic cells also form part of the corneal limbus. Additionally, limbal mesenchymal stromal cells (L-MSCs) are present^3^, which are hypothesised to assist in the maintenance of the LESC niche^4^. These cells are phenotypically similar to human bone marrow-derived MSCs (hBM-MSCs) by their cell surface markers (CD73^+^, CD90^+^, CD105^+^, CD45^−^, CD34^−^) and their multipotency^3, 5, 6^. L-MSCs have also been demonstrated to have comparable immunosuppressive characteristics to hBM-MSCs^7, 8^, and have been isolated from human^3, 5, 6, 8^ and rabbit^7^ corneas. Nevertheless, these cells have not yet been profiled in the porcine cornea, which is a widely used model for the study of corneal diseases and therapies. Moreover, no specific cell surface markers have been reported to specifically indicate the L-MSC population in pigs. Noort and colleagues performed a large comparative study of MSCs derived from human or porcine bone marrow^9^. It was reported that the majority of anti-human antibodies did not cross-react with the porcine cells, making it difficult to characterise the MSCs. However, the authors described the lineage differentiation potential and immunomodulatory effects of porcine MSCs were similar to that found in human MSC^9^.

The literature suggests that a subpopulation of pericytes are also usually present in isolated MSC populations, as determined by CD146^+^ and CD34^−^ expression^10^. In addition, it is already known that CD146 positive cells are present in L-MSC populations^7^. However, at the same time, it was demonstrated that human L-MSC grown in serum-free medium (MesenCult-XF^TM^) display limited expression of CD146 when compared with L-MSC cultured in serum-supplemented medium^7^. Studies by Li and colleagues showed that human limbal niche cells were able to support the vascular network formed by HUVECs on a Matrigel-coated surface^11^, however the potential of porcine L-MSC to support angiogenesis has not yet been reported.

The aim of our study was to compare porcine limbal stromal cells (pLSCs), cultivated in either serum-supplemented medium or serum-free medium, with hBM-MSCs. The isolated cells were characterised by means of plastic adherence, cell surface phenotype, and multipotency. Furthermore, we compared the pericyte capabilities of pLSCs with hBM-MSCs to stimulate angiogenesis and stabilise tubulogenesis of endothelial cells. Finally, the ability of pLSCs to support porcine LESC (pLESC) expansion was assessed by co-culture on silk fibroin membranes as a possible carrier material for transplantation. Our results provide evidence for the MSC characteristics of pLSCs, but also exposes some differences between human and porcine L-MSCs.

## Results

### Characterisation of putative pLSC

**Figure 1:**
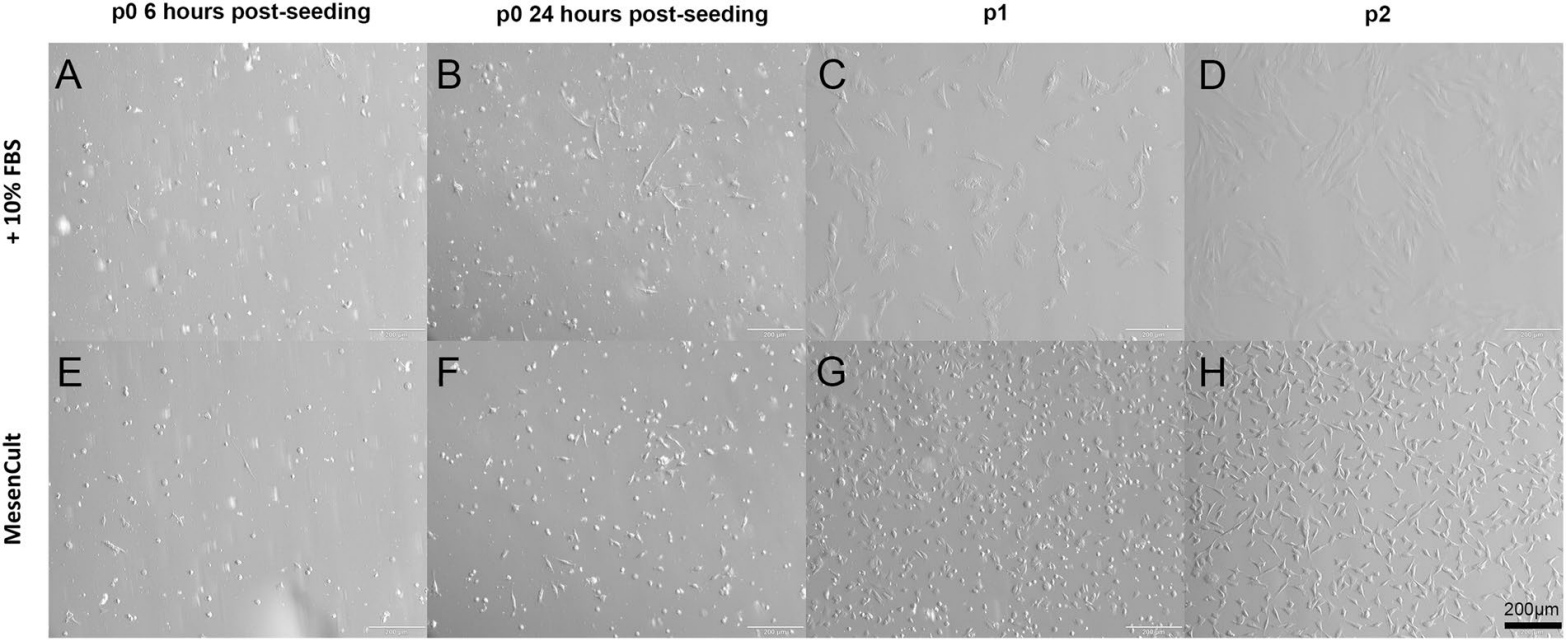
Morphology of adherent isolated pLSCs at 6 h (**A,E**) and 24 h post-seeding (**B,F**), and at passage 1 (**C,G**) and 2 (**D**,**H**) of culture. PLSC grown in serum-supplemented medium (top row) and serum-free conditions (bottom row) demonstrated adherence to tissue culture plastic and presented a spindle-shaped morphology. Scale bar = 200 μm.

The use of dispase treatment was found to retrieve the majority of pLESCs from the limbal region of the porcine ocular surface (Supplementary Figure S1). The first criterion for MSCs, as stated by the International Society for Cellular Therapy (ISCT)^12^ is their ability to adhere on tissue culture plastic under standard cell culture conditions. Six hours post-seeding, adherent cells were visualised on the flasks (Fig. 1A,E). These cells adopted a spindle-shaped morphology after 24 h in culture (Fig. 1B,F), and after the first passage, they assumed a more flattened morphology (Fig. 1C,D). When cultured under serum-free conditions (MesenCult^TM^-XF culture kit), cells adopted a smaller spindle-shaped morphology (Fig. 1G,H). Freshly isolated pLSCs reached 80–90% confluence within 7 days on average in both media.

**Figure 2:**
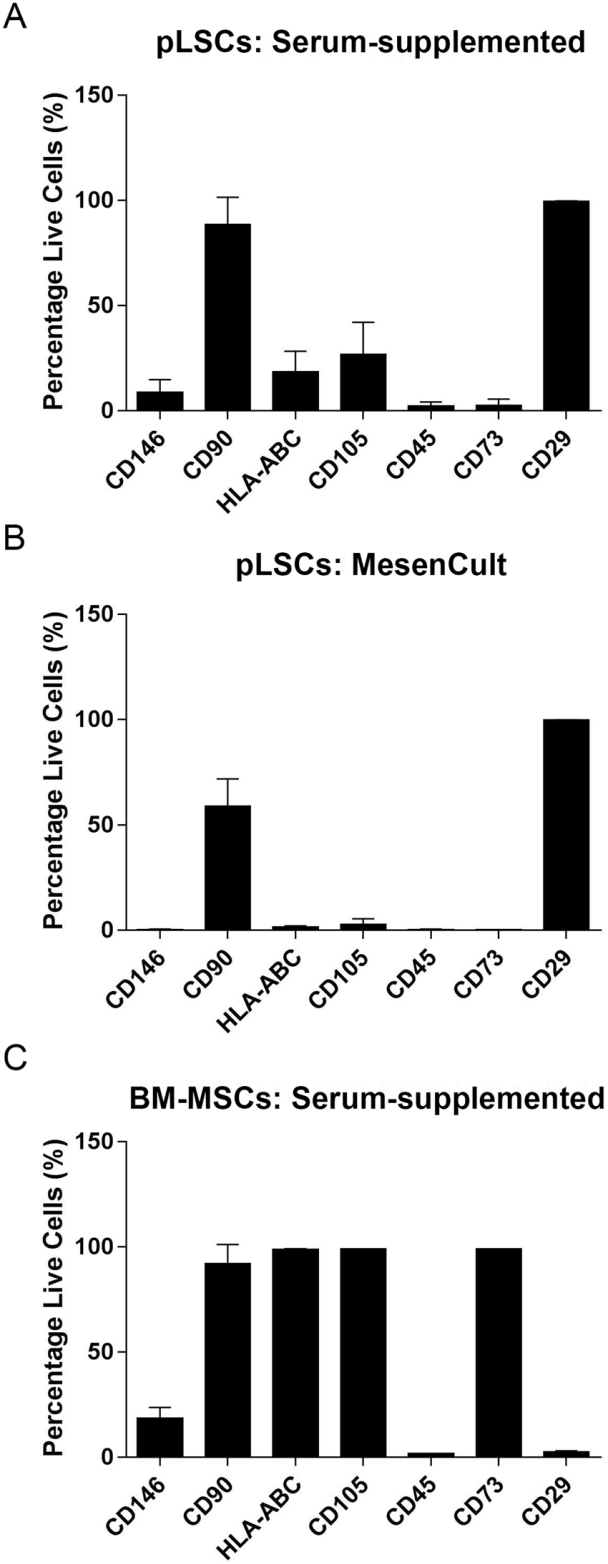
Phenotyping of pLSCs by flow cytometry. PLSCs grown in medium containing 10% FBS (**A**) or in serum-free MesenCult^TM^-XF medium (**B**). HBM-MSCs were included as a control (**C**). Data is shown as mean ± standard deviation. Values were pooled from three biological replicates performed in technical duplicates.

**Figure 3:**
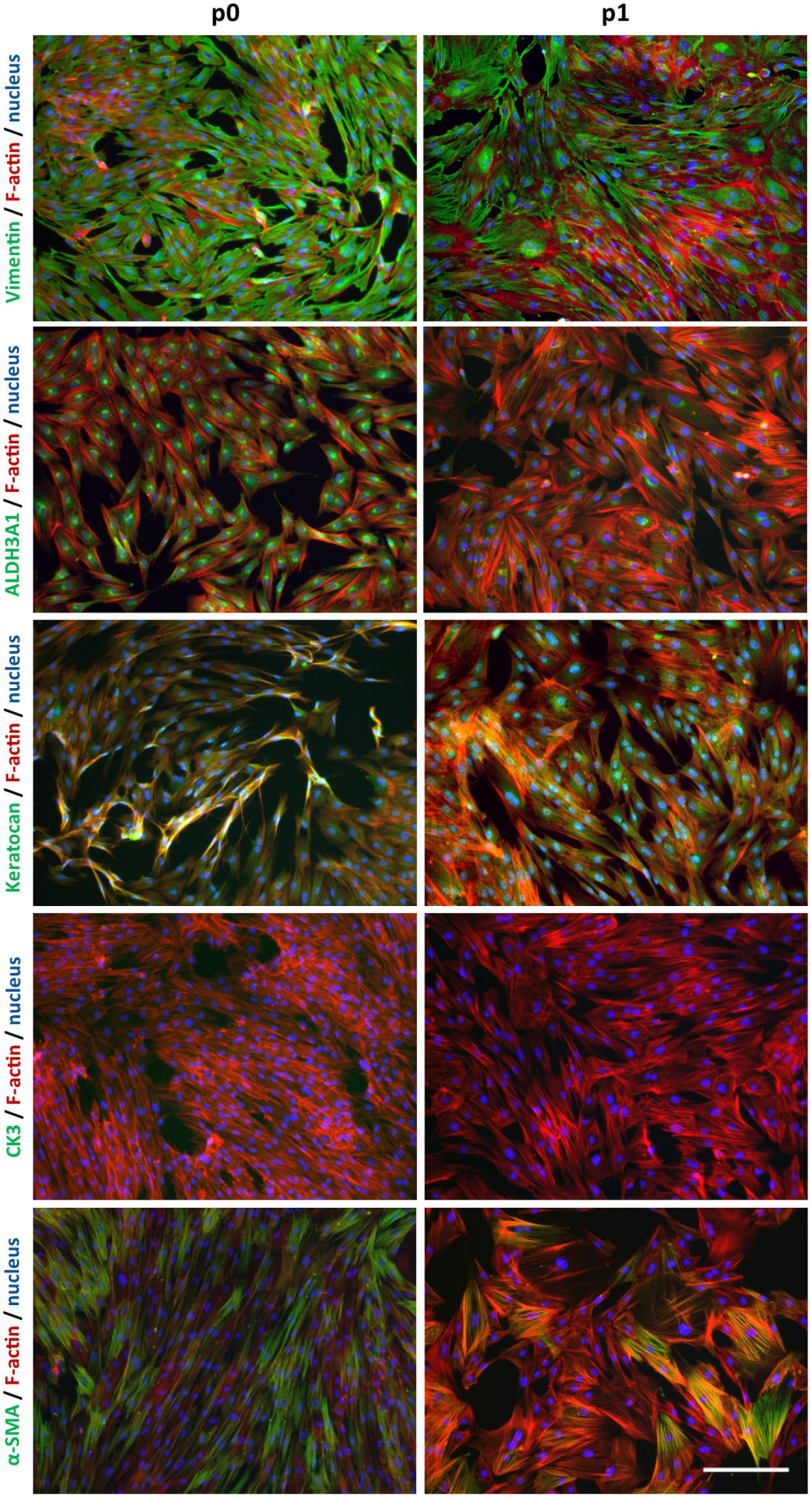
Phenotype of pLSCs cultured in serum-supplemented medium at passage 0 and passage 1. PLSC at passage 0 (p0) and passage 1 (p1) were stained for a variety of markers, including keratocyte markers (ALDH3A1 and keratocan), myofibroblastic markers (α-SMA), mesenchymal stromal cells (vimentin) and epithelial cells (cytokeratin 3). Keratocyte markers showed a decreased expression after one passage, while mesenchymal markers kept constant. No epithelial contamination was observed by the lack of cytokeratin staining. Some α-SMA positive cells could be seen at both p0 and p1. Scale bar = 200 µm.

According to the ISCT criteria^12^, cells must have the following profile to be called MSCs. Cells should be ≥95% positive for CD73, CD90 and CD105, and ≤2% negative for CD34, CD45, CD14 or CD11b, CD79α or CD19, and HLA-DR. Due to the lack of porcine species-specific antibodies, the majority of phenotyping in this study was carried out using anti-human antibodies, with the exception of CD29. Positive and negative expression of antibodies is detailed in Supplementary Table S1. As seen in Fig. 2A, pLSCs cultured with 10% FBS were 99.72 ± 0.14% positive for CD29, 88.65 ± 12.24% positive for CD90, 26.84 ± 14.40% positive for CD105, 18.47 ± 9.37% positive for HLA-ABC, 8.86 ± 5.67% positive for CD146, and negative for CD45 (2.26 ± 1.81%) and CD73 (2.53 ± 2.88%). PLSCs grown in serum-free MesenCult^TM^-XF medium showed a similar phenotype and were positive for CD29 (99.76 ± 0.14%), but with decreased CD90 expression (58.65 ± 12.00%), and negative for CD146, CD105, and HLA-ABC (Fig. 2B). Similar to serum-supplemented cultures, pLSC cultured in MesenCult^TM^-XF were also negative for CD45 and CD73 (Fig. 2B). Bone marrow-derived MSCs (Fig. 2C) and hL-MSC (Supplementary Figure S2) were used as positive control and expressed all characteristic MSC markers. Porcine CD29 did not cross-react with human BM-MSC. In addition, the expression of various mesenchymal, myofibroblast, keratocyte, and epithelial markers were investigated in pLSC cultures grown in serum-supplemented medium. PLSC cultures were positive for vimentin, ALDH3A1, Keratocan, and alpha smooth muscle actin, while were negative for cytokeratin 3 (Fig. 3) at both passage 0 and passage 1.

**Figure 4:**
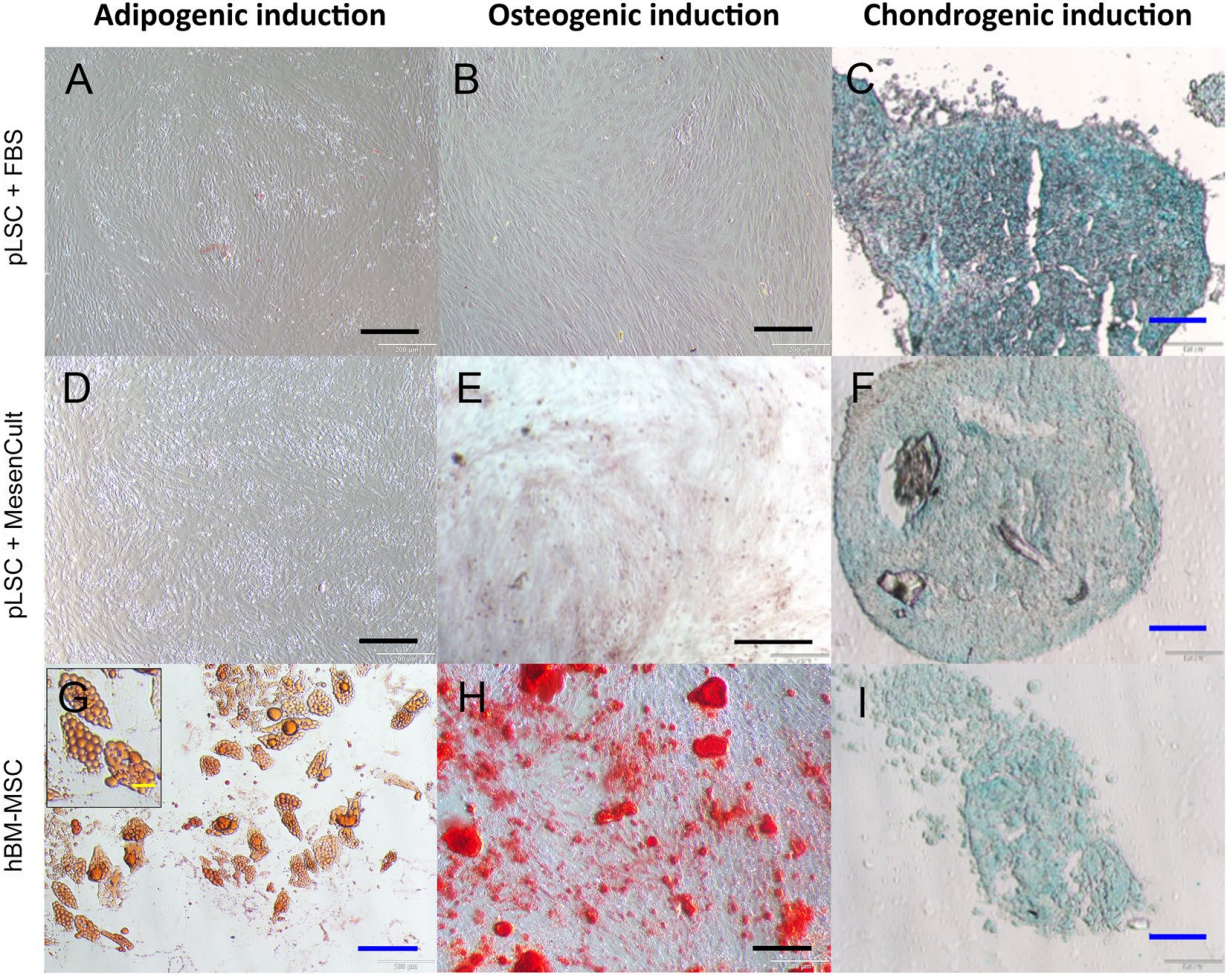
Differentiation of pLSCs and hBM-MSCs into adipogenic (**A,D,G**), osteogenic (**B,E,H**), and chondrogenic (**C,F,I**) lineages. Adipogenic and osteogenic differentiation occurred in BM-MSC cultures but not in pLS, as confirmed by Oil Red O and Alizarin Red staining respectively (left and center column). Chondrogenesis differentiation was observed in all conditions as assessed by Alcian Blue staining (right column). Black scale bar = 200 μm, blue scale bar = 100 μm, yellow scale bar = 20 μm.

The third criterion to validate cells as MSCs is their multipotency towards the adipogenic, osteogenic, and chondrogenic lineages. Control human BM-MSC cultures displayed typical adipogenic, osteogenic, and chondrogenic potential (Fig. 4G-I). PLSCs did not differentiate into adipocytes, as no lipid droplets could be seen under the bright field microscope after Oil Red O staining (Fig. 4A,D). Serum-free culture conditions did not improve the differentiation potential of pLSCs. Clear differentiation towards the osteogenic lineage was only seen in BM-MSC cultures (Fig. 4H), while pLSC grown in both medium conditions were clearly negative (Fig. 4B,E). Differentiation towards the chondrogenic lineage was observed in all three cell groups tested (Fig. 4C,F,I).

### PLSCs support formation of a vascular network in 3D hydrogel models

**Figure 5:**
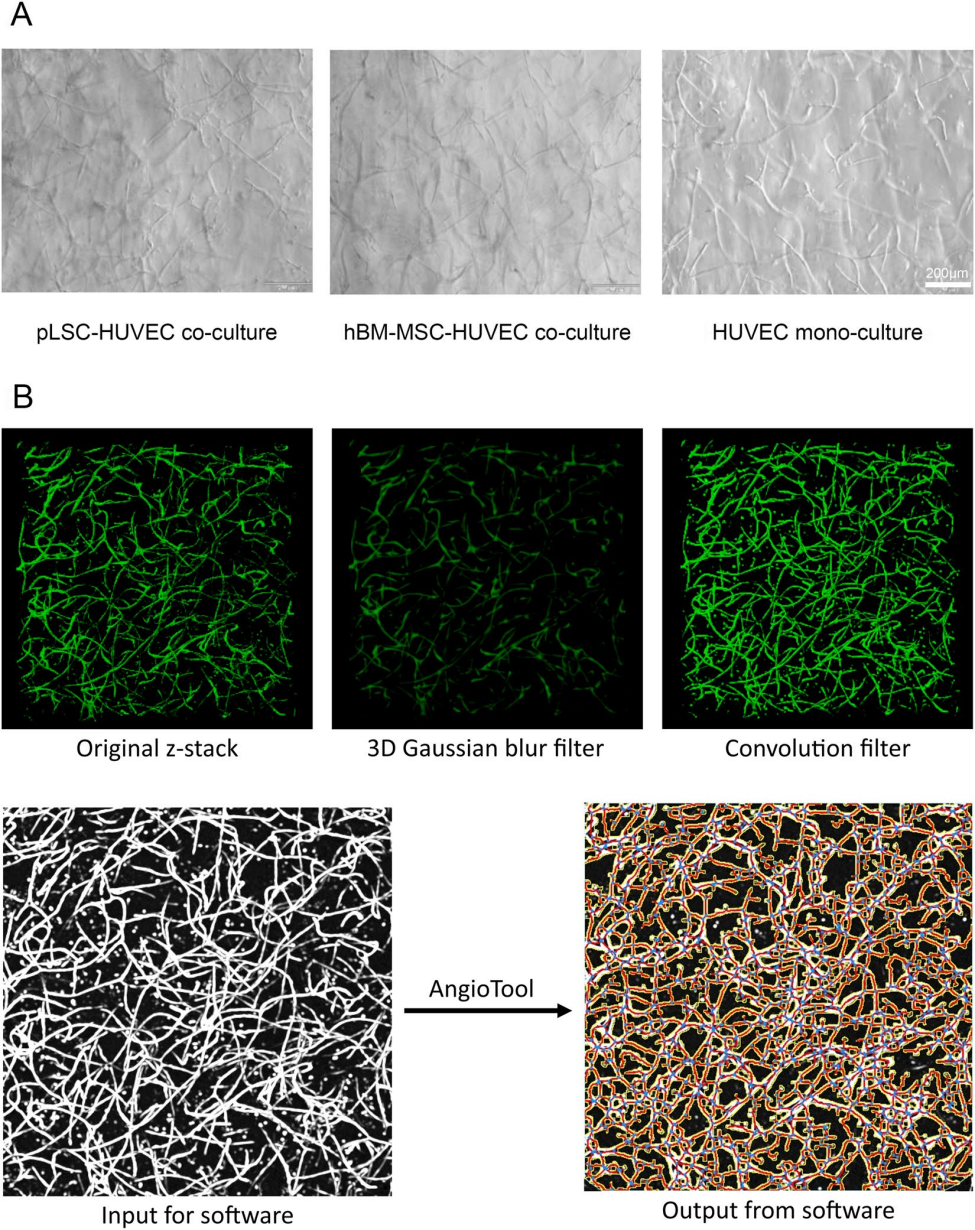
Analysis of pLSC-HUVEC network formation. Light microscope images of HUVEC co-cultures in starPEG-heparin hydrogels at day 7 (**A**). Scale bar = 200 μm. Sequential steps in the quantification procedure of HUVEC network formation using Fiji (Image J) and AngioTool, exemplary images (**B**).

**Figure 6:**
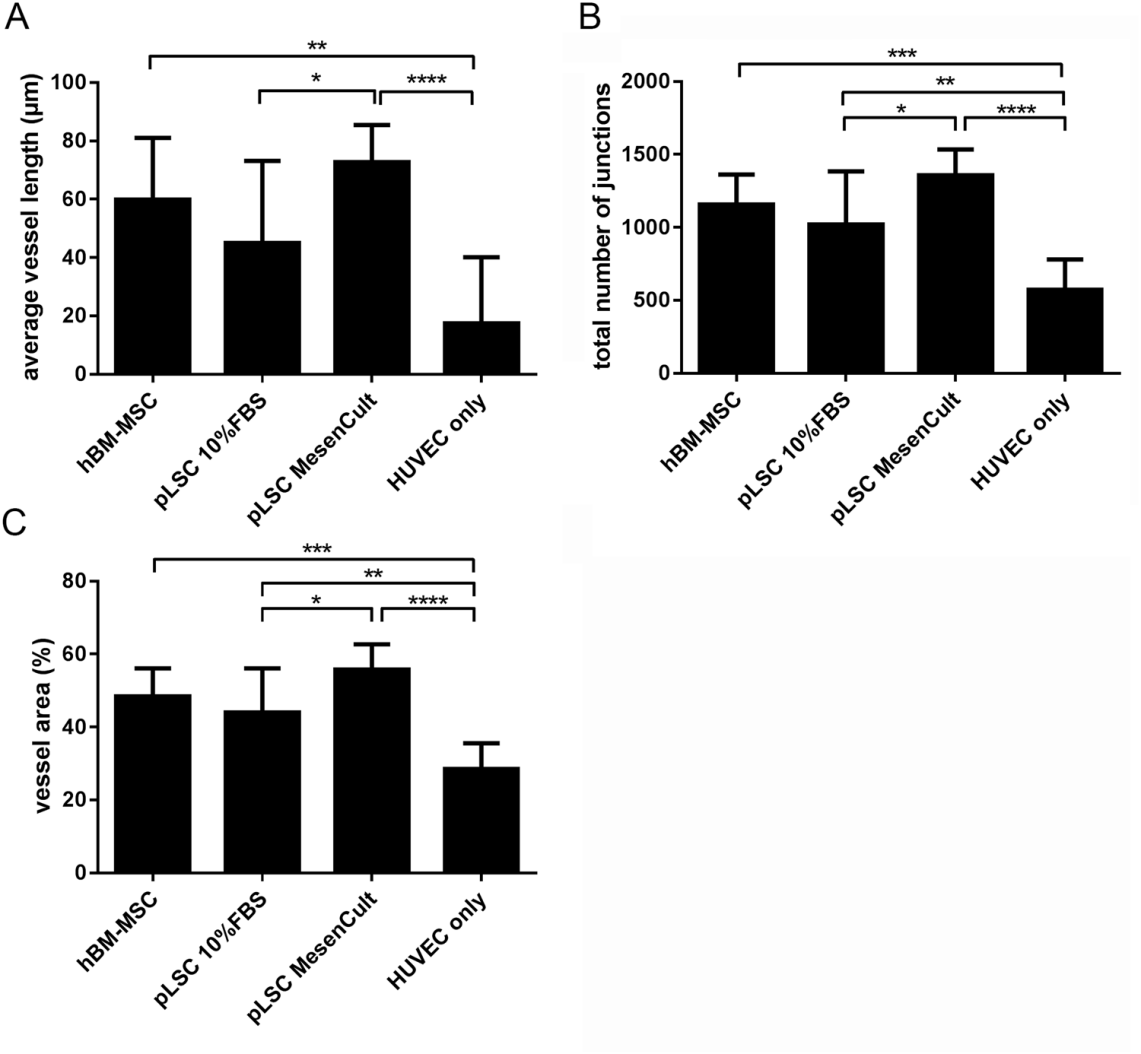
Angiogenesis enhancement of pLSCs or hBM-MSCs on HUVEC tube formation in starPEG-heparin hydrogels. Cultures were stained for CD31 after 7 days of culture, and confocal z-stacks were analyzed with AngioTool software. Parameters explored were: average vessel length (**A**), total number of junctions (**B**), and vessel area (**C**). Graphs show mean ± standard deviation. Data was pooled from three independent experiments performed in triplicate. Statistical significance was calculated using one-way ANOVA and Tukey’s multiple comparison test. *p ≤ 0.05, **p ≤ 0.01, ***p ≤ 0.001, ****p ≤ 0.0001.

The capacity of pLSC, grown in both serum-supplemented and serum-free medium to support and stabilise vascular network formation by HUVECs was investigated. To achieve angiogenesis network formation using HUVECs, soft gels were used. The storage modulus of the hydrogels utilised for angiogenesis studies was 388.5 ± 55.8 Pa (mean ± SD). HBM-MSCs were used as positive controls as they have been previously shown to stabilise the capillary-like structure of HUVECs formed within the gels^13^. Bright field images of co-cultures and mono-cultures in the starPEG-heparin hydrogels are shown in Fig. 5A, along with exemplifying pictures of each step of network quantification performed by the software (AngioTool) (Fig. 5B). It was found that the addition of supporting cells enhanced tubulogenesis (Fig. 6). The average vessel length was significantly increased in all co-cultures, 59.76 ± 21.3 μm, 44.98 ± 28.14 μm, and 72.68 ± 12.79 μm, for hBM-MSCs, serum-supplemented pLSCs, and serum-free pLSCs respectively, when compared with HUVEC mono-cultures (17.40 ± 22.72 μm) (Fig. 6A). The total number of junctions (branching of vessels) was also enhanced in all co-cultures when compared with HUVEC mono-cultures. HUVEC mono-cultures had 573.10 ± 208.5 total junctions, whereas hBM-MSCs, serum-supplemented pLSCs, and serum-free pLSCs had 1157.00 ± 204.90, 1018.00 ± 365.90, and 1357.00 ± 177.5 total junctions, respectively (Fig. 6B). The percentage vessel area, which is the fraction occupied by vessels from the total sampled volume, was increased in all co-cultures (Fig. 6C). HUVEC mono-cultures represented 28.49 ± 7.05% of the total gel volume, whereas hBM-MSCs, serum-supplemented pLSCs, and serum-free pLSCs represented 48.40 ± 7.73%, 43.97 ± 12.14%, and 55.78 ± 6.94%, respectively (Fig. 6C). In summary, HUVEC networks in co-culture had longer vessels with more branch points, occupying more space.

### PLSC support the culture of pLESC on silk fibroin membranes

**Figure 7:**
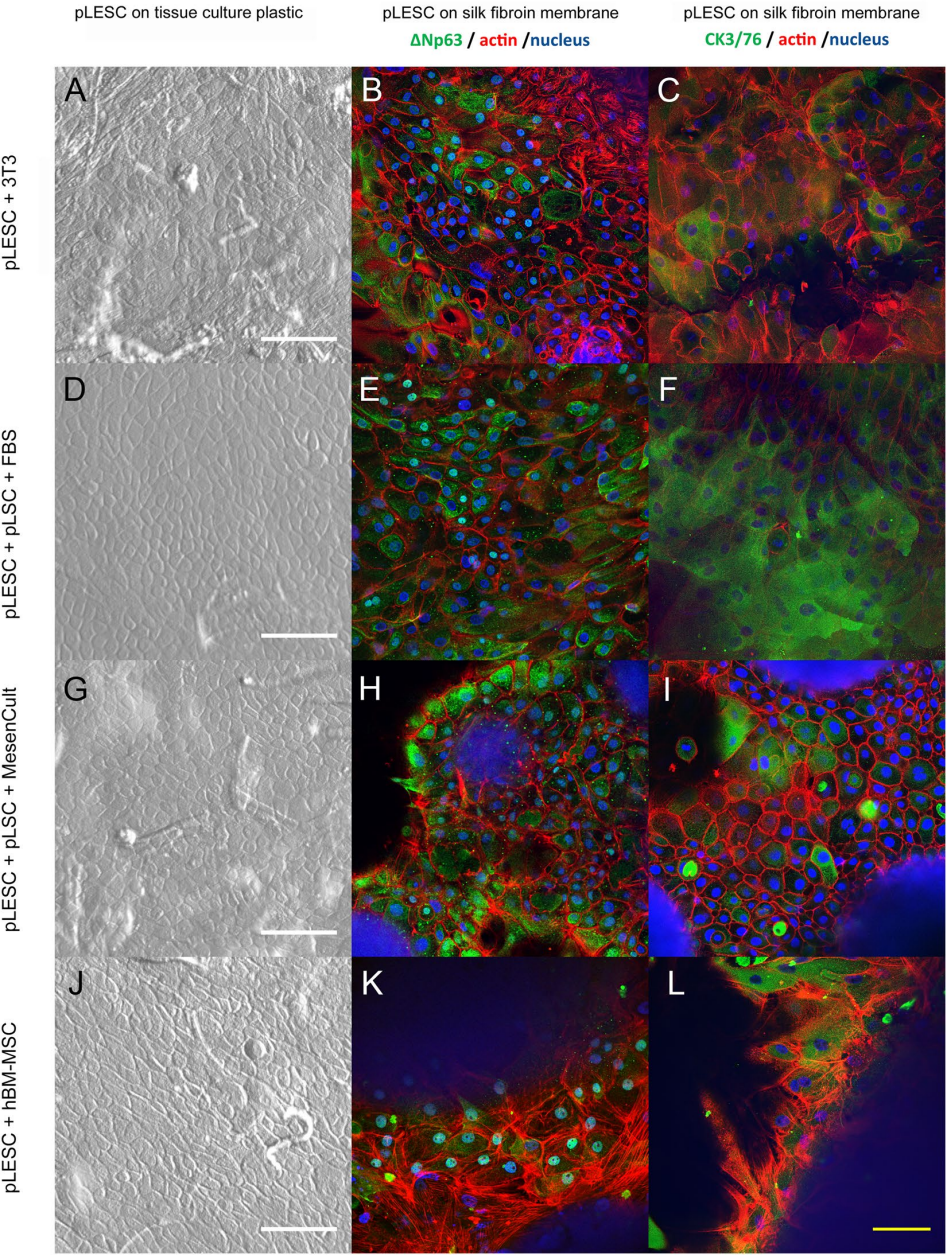
Capacity of pLSCs, hBM-MSCs and 3T3s to support pLESC growth. PLESCs were cultured on silk fibroin membranes with growth-arrested murine 3T3 fibroblasts (**A–C**), pLSCs grown in serum-containing (**D–F**) or serum-free media (**G–I**), and hBM-MSCs (**J–L**). Typical cobblestone morphology could be seen under the bright field microscope (left column). ΔNp63-positive cells (center column) and cytokeratin (CK) 3/76 positive staining (right column) were visible. White scale bar = 200 μm, yellow scale bar = 300 μm.

PLSCs (grown either with 10% FBS or MesenCult^TM^-XF) were growth arrested with Mitomycin C and used as feeder cells for the culture of pLESCs. PLESCs were grown on growth-arrested 3T3 murine fibroblast cells as a control, as these are the “gold standard” for pLESC sheet generation. PLESCs were also cultured on growth-arrested hBM-MSCs as these cells have previously shown promising results supporting the growth of LESCs^14^. PLESCs cultured on pLSCs and on hBM-MSCs presented colonies with less defined edges, while pLESCs growing on 3T3s had well-defined colony boundaries; nevertheless, they all showed the typical cobblestone-shaped pattern. PLESC were first expanded on tissue culture plastic (Fig. 7A,D,G,J) and subsequently cultured on silk fibroin membranes, supported with 3T3s, hBM-MSCs and pLSCs, respectively (Fig. 7B,C,E,F,H,I,K,L). Attachment and growth of pLESC on silk fibroin membranes was achieved with all feeder cell types, obtaining a tight cobblestone pattern. PLESCs were positive for the putative LESC marker ΔNp63, which indicates that the cells maintained their stem cell characteristics (Fig. 7B,E,H,K). PLESCs cultured on hBM-MSCs predominantly displayed ΔNp63 in the nucleus of the cells (Fig. 7K), while pLESC culture on 3T3s or pLSCs showed cytoplasmic staining (Fig. 7B,E,H). On the other hand, disperse positive staining for cytokeratin 3/76 could be seen throughout the samples with all feeder cells (Fig. 7C,F,I,L), indicating corneal differentiation.

## Discussion

In the human corneal limbus, there is a population of cells with mesenchymal characteristics, similar to the MSCs found in the bone marrow^3, 5, 6^. These cells are hypothesised to assist in corneal wound healing and regeneration^11^, and therefore are important to consider when studying diseases of the ocular surface. Although many pre-clinical studies using porcine models have been performed to improve our understanding of corneal disorders, the characterisation of porcine cell populations in this area has been lacking. Therefore, an evaluation of porcine stromal phenotype is necessary to reliably predict pre-clinical data on corneal therapies. MSCs have a great potential for regenerative therapies as they have immunosuppressive activity, multi-lineage capacity and homing role for stem cells. MSC derived from the limbus could be utilised in applications as feeder cells for the *ex vivo* expansion of LESCs, substituting the standard murine 3T3 feeder cells. In addition, these MSC could be utilised as a cellular therapy for restoration of the limbal or corneal stroma, and as such, have direct benefits as a therapeutic tool within the area of both human and veterinary medicine. We here investigated the MSC characteristics of LSCs isolated from porcine tissue and demonstrate their capacity for angiogenic and LESC support.

Evidently, the “absence” of porcine-specific antibodies renders phenotyping by flow cytometry difficult. As described by others^9, 15^, anti-human CD90 and CD146 antibodies showed species cross-reactivity, while CD73 did not. In our study, anti-human CD105 and HLA-ABC antibodies did cross-react to a certain extent with the isolated pLSCs. This contradicts findings from Rozemuller and colleagues^15^, who found these markers to be under detection limit in porcine BM-MSCs, although the antibodies were purchased from a different source. The culture of human L-MSCs under serum-free conditions has been reported to show a decrease in CD146 expression^5^. Likewise, this phenomenon was also observed for pLSCs in our study. CD146 is a cell adhesion molecule expressed in a wide variety of cell types, including vascular endothelial cells, pericytes, smooth muscle cells and MSCs, all of which are found in the limbal niche. Moreover, CD105, also named Endoglin, is part of the TGF-β receptor complex and plays an important role in angiogenesis^16^. A decrease in CD146 and CD105 expression was visualised for pLSCs cultured in MesenCult^TM^-XF when compared with serum-supplemented medium. HLA-ABC expression for pLSCs was also downregulated during culture in serum-free conditions, which indicates the presence of a less immunosuppressive phenotype. As previously described^5, 7^, the decrease in CD146 expression in human L-MSCs cultured in MesenCult^TM^ XF did not diminish their multipotency. On the contrary, the differentiation potential of serum-free cultured hL-MSCs was higher than that of L-MSCs grown in serum-supplemented medium. Interestingly, others report that higher CD146 expression is linked to increased multipotency^17^. However, this could not be seen in our study, as pLSC differentiation could be seen in both culture conditions for chondrogenesis induction, but not for adipogenesis or osteogenesis. This was unexpected since the pLSCs used in this work display a comparative phenotype with pBM-MSCs. In our experiments, a decrease in CD105 expression was seen pLSCs cultured in serum-free medium. This was also observed by others for human bone marrow MSCs using serum-free medium (from a different supplier)^18^. However, no decrease of CD105 for human L-MSCs grown in serum-free medium has been reported^5^. Unfortunately, no specific set of markers for the characterisation of porcine MSCs is yet available, however the present phenotypic results closely compare with those previously reported for porcine BM-MSCs^9^.

Angiogenesis, the process of vessel formation, is important for tissue engineering and microenvironment emulation (especially in perivascular stem cell niches, such as the corneal limbal stem cell niche). It is a complex and highly regulated process involving signaling molecules, cell-cell interactions and cell-matrix interactions. Pericyte-endothelial cell interactions can be studied in three dimensional hydrogels^13^. Most angiogenesis tests focus on the endothelial capacity of forming capillary-like structures on a two dimensional surface coated with extracellular matrix components such as Matrigel^®^ or Geltrex^TM^. However, networks formed with these approaches do not last longer than 24 hours and therefore cannot interpret the pericyte capabilities of a cell. The hydrogel platform used in this study overcomes the above mentioned limitations: it provides three dimensional information with enough stability for a longer test duration. In addition, the hydrogel is able to mimic cell-instructive ECM properties via the incorporation of cell-adhesive ligands. Briefly, the hydrogel used here consists of four-arm PEG (starPEG) and the highly sulphated glycosaminoglycan heparin. The PEG component possesses cleavable sequences for matrix metalloproteases (MMPs) secreted by cells and a thiol group end functionality. The thiol groups react with the maleimide groups of chemically modified heparin via Michael-addition forming a covalently cross-linked network^19^. Furthermore, soluble factors which are typically positively-charged can interact with the negatively-charged heparin via electrostatic forces and be delivered to the embedded cells. Moreover, cell-adhesive peptides such as the integrin recognition motif (RGD) can be covalently bound to the heparin-maleimide conjugates prior to gel network formation. In our set-up, isolated stromal cells showed supporting capacities of HUVEC angiogenesis over a 7 day period, by increasing the average vessel length, volume occupied by the vessels, and the number of total junctions. These findings are in accordance with studies from Li and colleagues^11^ who similarly showed that cells from the limbus have the capacity to support angiogenesis. By culturing cells isolated from the human corneal limbus (which the authors called niche cells) on Matrigel^®^-coated plates and subsequently into 3D Matrigel^®^ spheres, they showed that the niche cells adopted a pericyte-like phenotype. These cells supported a HUVEC network for up to 5 days and showed direct contact of the limbal cells with the endothelial cells, similar to *in vivo* conditions, where pericytes and endothelial cells cooperatively share their basement membrane. Interestingly, in our work, the serum-free cultured pLSCs, which had decreased CD105 and CD146 expression, displayed significantly higher pericyte capacity than serum-supplemented pLSCs.

LSCD can be treated by the transplantation of autologous or allogenic LESCs. The state-of-the-art procedure is to obtain small limbal biopsy, allow the explant to expand *ex vivo* on a feeder layer of growth-arrested murine fibroblasts and transplant the cells onto the patient’s ocular surface using human amniotic membrane (hAM) as a carrier^20^. This technique, although proven to be successful, poses risks of disease transmission in different stages. Diseases can have a xenogenic origin from the feeder cells, from animal serum used during culture, or from the origin of the hAM^21^. In recent years, other cells sources have been investigated and examined as alternatives to the currently used feeder cells, to minimise these risks^3, 5–7^. While BM-MSCs have been shown to support growth of LESCs^14^, cells found in the epithelial stem cell niche have arisen as a logical alternative as they (most probably) secrete cytokines and growth factors which signal to the epithelial stem cells to adopt a proliferative but not differentiating phenotype. Previous studies suggest that L-MSCs are suitable for cell expansion prior to transplantation^5, 7, 22^.

Previous studies show that human LESCs attach to silk fibroin membranes to a similar extent as for tissue culture plastic^23^. Higa *et al*. have cultured rabbit LESCs with 3T3 cells on porous fibroin membranes prepared by mixing aqueous fibroin solution with PEG^24^. Moreover, we have previously reported the growth of hLESCs on pure fibroin freestanding membranes in the presence of irradiated 3T3 cells or L-MSCs^23, 25^ with comparable cell attachment to tissue culture plastic and a clinically relevant phenotype (with basal cells expressing p63 and cells from upper layer expressing cytokeratin 3). In our study, the isolated pLSCs were used as an alternative to murine fibroblasts as feeder cells for the expansion of pLESCs. These cells supported the growth of the epithelial progenitors in a similar way as the “gold standard” 3T3 cells. In a similar way, previous studies showed that irradiated fibroblasts isolated from the limbus better supported the growth of epithelial progenitors when compared to fibroblasts isolated from the central cornea or from the sclera^26^.

The isolated LSCs appear to be an alternative to the “gold standard” 3T3 cells as feeder cells for the expansion of LESCs. Although we discovered that these cells are not typical MSCs, as they do not fulfil the minimal criteria proposed by the ISCT, and thus, they might not possess immunosuppressive properties as shown in human L-MSC. Nevertheless, they support the growth and maintenance of LESC properties. As seen in the bright field images of cultured pLESCs on the different feeder cells, the growth-arrested cells are pushed aside and eventually lift off as the epithelium proliferates. For long culture periods (more than 14 days) this can be a potential issue as the epithelium may lack the growth signals mediated by the feeder cells as an *in vitro* microenvironment. To overcome this, a possible solution would be to seed the feeder cells on one side of a membrane (such as fibroin), and the LESCs on the other. Diffusion of nutrients and signalling molecules should not be affected as the membranes are known to be permeable to small molecules^23^.

Based on the above considerations, we have evaluated strategies for the isolation and culture of pLSCs and performed an extensive MSC phenotype profile, as well as examined their differentiation abilities. Our results demonstrate marked differences in MSC phenotype between human and porcine LSCs, but provide evidence of their angiogenesis and LESC supporting properties. However, it should be noted that the multipotent potential of pLSCs was limited.

## Methods

### Primary cell isolation

Porcine eyes were obtained from a local slaughterhouse. The eyes were washed twice with phosphate buffered saline (PBS; Sigma-Aldrich, Steinheim, Germany), treated with iodine for 4 min under agitation and rinsed three times with PBS for 2 min. Using a scalpel and scissors under sterile conditions, the cornea was excised, removing the iris and as many pigmented areas as possible. The central cornea was removed and the remaining limbus was cut into four pieces and incubated with 2.5 mg/ml dispase (Life Technologies, Darmstadt, Germany) at 37 °C for 1 h. Limbal epithelial sheets were collected by scraping the limbal surface with a scalpel blade and pipette tip using a stereo microscope, the collected cells were pooled and centrifuged at 1000 rpm for 5 min. The cells were then resuspended in epithelial medium (described below) and seeded with growth-arrested feeder cells. 3T3s were seeded at a density of 3 × 10^4^ cells/cm^2^ and stromal cells (pLSCs or hBM-MSCs) at a density of 2 × 10^4^ cells/cm^2^. To obtain limbal stromal cells, the pieces of remaining corneal limbus were cut into smaller pieces and treated with 1 mg/ml collagenase (Life Technologies) for 48 h at 37 °C. Then, cells were collected and centrifuged at 1000 rpm for 5 min and seeded into culture flasks with limbal stromal medium (described below). To determine the efficiency of epithelial cell harvest post-dispase treatment, corneo-scleral pieces were fixed after cell isolation in 4% paraformaldehyde (PFA) overnight at 4 °C. Samples were then embedded in paraffin after a series of dehydration steps in increasing ethanol concentrations followed by immersion in xylene. 8 µm-thick sections were obtained with a microtome (Leica model RM2125RT), deparaffinised and mounted with mounting medium containing DAPI (ab104139, Abcam). Images were taken using a fluorescent microscope (Olympus IX8).

### Cell culture and medium

Epithelial medium consisted of a 1:3 mixture of Dulbecco’s Modified Eagle’s Medium (DMEM; Life Technologies) and Hams F12 medium (Life Technologies), supplemented with 10% foetal bovine serum (FBS; Hyclone Thermo Scientific, Schwerte, Germany), 1% penicillin/streptomycin (PS; Life Technologies), 10 ng/mL epidermal growth factor (Sigma-Aldrich), 1% v/v non-essential amino acids (Life Technologies), 2 mM L-glutamine, 6.8 µg 3,3,5-triiodo-L-thyronine sodium salt (T3), 180 µM adenine, 5 µg/mL transferrin, 0.4 µg/mL hydrocortisone, 1 µg/mL bovine insulin and 10 ng/ml Cholera toxin (all Sigma-Aldrich). The medium utilised for limbal stromal cell cultivation was DMEM/F12 (Life Technologies) supplemented with 10% FBS and 1% PS, or MesenCult^TM^-XF (StemCell Technologies, Köln, Germany) xeno-free culture kit and serum-free medium supplemented with 2mM L-Glutamine and 1% PS. NIH/3T3 murine fibroblasts were cultured in high glucose DMEM (Life Technologies) supplemented with 10% FBS and 1% PS. The HUVEC medium utilised was PromoCell Endothelial Cell Growth Medium (Heidelberg, Germany). HBM-MSCs were grown with DMEM (Life Technologies) supplemented with 10% FBS (Biochrom AG, Berlin, Germany) and 1% PS. 10 μg/ml Mitomycin C (Sigma-Aldrich) in medium was used to growth-arrest cells, incubating at 37 °C for at least 2 h for 3T3s and 4 h for pLSCs grown in MesenCult^TM^-XF. Cell cultures, both 2D cultures and hydrogel-embedded cells, were fed every two days with fresh medium. Images of cultures were taken regularly under the bright field microscope (Olympus IX73, Hamburg, Germany).

### Flow cytometry

The phenotype of pLSCs and hBM-MSCs was assessed by flow cytometry using a MACSQuant Analyzer (Milteny Biotec, Bergisch Gladbach, Germany) and results were analysed using FlowJo software (Tree Star Inc.). HBM-MSCs were used as positive control. After harvest, cells were resuspended in MACS running buffer to a concentration between 5 × 10^4^ to 1 × 10^6^ cells per 100 μl. The panel of antibodies used was the following: mouse anti-human CD146-phycoerythrin (PE; BD Biosciences, Heidelberg, Germany), CD90-fluorescein isothiocyanate (FITC; BD Biosciences) and HLA-ABC-allophycocyanin (APC; eBioscience, Frankfurt am Main, Germany); CD105-PE (eBioscience), CD45-FITC (Miltenyi, Bergisch Gladbach, Germany) and CD73-APC (eBioscience). Purified CD29 (raised in mouse against porcine antigen) from BD Biosciences was incubated for 1 h at 4 °C, washed with MACS running buffer, centrifuged for 5 min at 300 g and an Alexa488-coupled secondary antibody (goat anti-mouse; Life Technologies) was added and incubated for 30 min at 4 °C. Primary and secondary antibodies were added at a volume of 1 μl per sample. Fluorophore-coupled antibodies were added at concentrations recommended by the manufacturer and were incubated at 4 °C for at least 20 min, washed with MACS running buffer, centrifuged for 5 min at 300 g, supernatant removed and resuspended in 200 μl of fresh MACS running buffer. Samples were tested in duplicate. The number of events recorded was 4 × 10^4^ using a high flow rate.

### Differentiation assay

PLSCs were tested for multipotency under adipogenic, osteogenic and chondrogenic conditions. Human BM-MSCs were used as positive controls. For adipogenic induction, 5 × 10^4^ cells per well (24-well plates) were seeded with standard MSC medium and left to attach for 24 h before changing into adipogenic induction medium containing 1 μM dexamethasone, 500 μM isobutyl methylxanthine (IMBX), 100 μM indomethacin and 1 μg/ml insulin. Osteogenic induction was performed similarly but 2 × 10^4^ cells per well were seeded. Osteogenic induction medium contained 50 μM 2-phospho ascorbic acid, 10 mM β-glycerol phosphate and 100 nM dexamethasone. Medium was changed completely every 3–4 days. For chondrogenesis differentiation, 5 × 10^5^ cells were seeded in 15-ml tubes, centrifuged 5 min at 300 g, supernatant removed, chondrogenic induction medium added (1 ml), mixed well and centrifuged a second time. Chondrogenic induction medium used was StemPro® Chondrogenesis Differentiation Kit (Life Technologies). Every 4 days, 500 µl was removed and 500 µl fresh medium was added carefully to avoid disturbing the pellet/micromass. Cells were cultured for a period of 21 days and bright field images were taken periodically. If not otherwise stated, all steps for staining were performed at room temperature (21 °C). Adipogenesis differentiation was assessed by Oil Red O staining, which stains neutral lipids. Cells were fixed with 4% PFA for 15 min and rinsed with double distilled water (ddH_2_O). Then, cells were incubated for 5 min with 60% isopropanol. Upon removal of the isopropanol, Oil Red O working solution was added and incubated for 5 min. Solution was removed and rinsed with ddH_2_O until solution rinsed off clear (around 4 washes). Working solution was prepared by mixing 3 parts of Oil Red O stock solution (3 mg/ml of Oil Red O powder, Sigma-Aldrich, in 99% isopropanol) with 2 parts of ddH_2_O, incubated for 10 min and filtered through Whatman filter paper. Osteogenesis was assessed by Alizarin Red S (Sigma-Aldrich) staining. Cultures were fixed with 4% PFA for 15 min and washed with ddH_2_O. Alizarin Red staining solution (2 g of Alizarin Red powder in 100 ml ddH_2_O, pH adjusted to 4.1–4.3) was added and incubated for 45 min in the dark. Solution was removed and washed 4 times with ddH_2_O. Chondrogenic pellets were embedded in OCT, cryosectioned and stained using Alcian Blue (Sigma-Aldrich), which stains extracellular matrix components, such as weakly acidic sulfated mucins, hyaluronic acid and sialomucins, in dark blue. The samples were washed once for 5 min in PBS containing calcium and magnesium. They were then rinsed for 3 min in 3% acetic acid (Sgima-Aldrich) and incubated with 1% Alcian Blue in 3% acetic acid (with pH adjusted to 2.5) for 30 min at room temperature. Slides were rinsed briefly with 3% acetic acid, then rinsed with running tap water for 10 min and briefly with ddH_2_O water.

### Rheological measurements

Mechanical characterisation of hydrogels was carried out by rheology. Briefly, 67 μl hydrogels were cast in between microscopy slides coated with Sigmacote® (Sigma-Aldrich) and allowed to swell in PBS overnight. Prior to measurement gels were cut using a trephine into 8 mm in diameter cylinders. Storage modulus was obtained from the Rheometer (ARES LN2; TA Instruments, Eschborn, Germany). Frequency sweeps were performed at 25 °C with a shear frequency range of 10^−1^–10^2^ rad/s with strain amplitude of 2–7%.

### Immunostaining

All steps were performed at room temperature (21 °C) and mixing was performed by using a plate shaker at 250 rpm (Titramax 100, Heidolph, Schwabach, Germany). Both cells on tissue culture plastic and hydrogel-embedded cells were fixed with 4% PFA for 15 min. After two washes with PBS, gels were blocked with goat or donkey serum (Life Technologies) (5% serum in 0.5% Triton X-100 (Sigma-Aldrich) in PBS) for 2 h. Then, the primary antibody was added at 1:100 in 1% serum in 0.01% Triton X-100 in PBS and incubated overnight on the shaker protected from light. The next day, samples were washed with 1% serum in 0.05% Triton X-100 in PBS and kept at 4 °C. On the third day, gels were washed twice on the shaker at room temperature. The secondary antibody (Alexa488-conjugated, usually goat anti-mouse; Life Technologies, Oregon, USA) was added at 1:200 and Atto633- phalloidin (ATTOtec GmbH, Siegen, Germany) at 1:100 in 1% serum in 0.01% Triton X-100 in PBS and incubated overnight at room temperature and protected from light. The next day samples were washed with 1% serum in 0.01% Triton X-100 in PBS and kept at 4 °C. On the fifth day, gels were washed 2 times on the shaker at room temperature protected from light. Hoechst 33342 (Life Technologies) was added at 1:1000 in PBS and incubated for 30 min. Then samples were washed and imaged or stored in PBS at 4 °C and covered with Parafilm^®^ and protected from light. For 2D cultures, blocking, primary antibody, phalloidin and secondary antibody, and Hoechst incubation were 2 h each followed by three washing steps. Cells grown on silk membranes were directly immunostained against anti-human ΔNp63 (BioLegend, San Diego, USA) and cytokeratin 3/76 (Millipore, Pittsburgh, USA). Cells grown within hydrogels were stained for CD31 (BD Biosciences). Immunostained samples were imaged using an inverted confocal microscope (cLSM; SP5, Leica Microsystems, Wetzlar, Germany).

### Test for angiogenesis support

Hydrogels were prepared as described previously^19^. The heparin-maleimide conjugate was dissolved in PBS and functionalised with the integrin recognition sequence RGD (2 moles of RGD per mole of heparin). Growth factors were then added to the heparin: 5 μg/ml fibroblast growth factor 2 (FGF-2), stromal-derived factor 1 (SDF-1; both Miltenyi) and vascular endothelial growth factor (VEGF; Peprotech, Hamburg, Germany). Cells were harvested and resuspended in PBS and added to the heparin fraction. The concentration of HUVECs embedded was ten-fold higher than that of pLSCs or hBM-MSCs (1.2 × 10^5^ HUVECs and 1.2 × 10^4^ stromal cells per 20 μl gel). The starPEG-peptide conjugate was dissolved in PBS and sonicated for 3 min. The heparin suspension with cells and growth factors was mixed with the PEG solution at a 1:1 volume ratio by pipetting to obtain a 20 μl gel which was cast on a Sigmacote® coated microscopy slide. After some minutes to allow the polymerisation of the hydrogels, these were transferred into wells of a 24-well plate with 1 ml HUVEC medium. After seven days in culture, hydrogels were stained for CD31. Z-stacks were obtained via cLSM using the following parameters: long-range air 10x objective (NA 0.4), pinhole set at 1 AU, bidirectional scanning, step-size 2.52 μm and total volume of 456 μm.

To quantify network formation AngioTool was used^27^. First, the raw data from the z-stacks was treated to reduce background and noise using Fiji software (ImageJ, NIH). The Gaussian Blur 3D filter with a sigma of 2.0 was applied and subsequently the stack was convolved (using the default settings, normalizing the Kernel). The maximum intensity Z projection was obtained and saved to be used in the angiogenesis analysis. Images were opened in AngioTool and calibrated for pixel size (1 px = 3.03 µm). The software recognised the edges of the vessels automatically but the vessel diameter can be adjusted manually. The intensity threshold can be tuned if needed, depending on image quality. Particles smaller than 230 µm^2^ were removed from the analysis as those structures are not part of the vascular network. Once all settings were applied, the program was run, from which an image with the results overlapping the original image was obtained as well as a spreadsheet with the numerical values.

### Silk fibroin extraction

Silk fibroin was extracted from *Bombyx mori* cocoons as previously described^28^. Briefly, 0.7 cm x 0.7 cm pieces were cut from the cocoons and boiled for 1 h with sodium carbonate, rinsed twice with warm water and let dry for 12 h. The dried mat was dissolved in lithium bromide at 60 °C for 4 h. The solution was allowed to cool down and filtered through 0.80 μm and 0.20 μm syringe filters (connected in series) via an 18 G needle into a dialysis bag. The solution was dialysed by changing water at time points 1, 4, 7, 18, 30 and 48 h. After 72 h, the dialyzed solution was filtered again through 0.80 μm and 0.20 μm syringe filters (connected in series) without a needle into a plastic vessel and stored at 4 °C. Concentration was determined by mass/volume (w/v) percentage. Culture plates were covered with the fibroin solution with the volume needed to obtain a 4% w/v coating. The solution was let dry in the oven at room temperature with ventilation for 24 h and then transferred to a vacuum chamber at −80 kPa for 6 h in the presence of a beaker with distilled water. Prior to use, fibroin-coated plates were sterilised with 70% ethanol for 30 minutes and rinsed three times with PBS. Key steps of this procedure are shown in Supplementary Figure S3.

### Statistics

Statistical significance was determined using GraphPad Prism v6.0 software. One-way Analysis of Variance (ANOVA) was used to determine statistical significance, followed by a Tukey multiple comparisons test. Values were considered to be statistically significant when p < 0.05. Significance is highlighted as follows: *p < 0.05, **p < 0.01, ***p < 0.001, ****p < 0.0001.

## Electronic supplementary material

Supplementary Information
