# Supplementary material for: Limbal stromal cells derived from porcine tissue demonstrate mesenchymal characteristics in vitro
Source: Sci Rep. 2017 Jul 25;7:6377. doi: 10.1038/s41598-017-06898-2 (PMC5527094; doi:10.1038/s41598-017-06898-2)
Supplement: Supplementary file 1 — Supplementary Information [file 41598_2017_6898_MOESM1_ESM.pdf]

Supplementary Information

Limbal stromal cells derived from porcine tissue demonstrate mesenchymal characteristics *in vitro*

Julia Fernández Pérez [1, 2], Marcus Binner [1], Carsten Werner [1, 3], Laura J. Bray [1, 4]

[1]*Leibniz Institute of Polymer Research Dresden, Max Bergmann Center of Biomaterials Dresden, Center of Regenerative Therapies, Hohe Straße 6, Dresden, Saxony 01069, Germany*

[2]*Current address: Trinity Centre for Bioengineering, Trinity Biomedical Science Institute, Trinity College Dublin 2, Ireland*

[3]*Dresden University of Technology, Dresden, Saxony 01069, Germany*

[4]*Queensland University of Technology (QUT), Kelvin Grove, Queensland 4059, Australia*

Corresponding author:

Laura J. Bray

Queensland University of Technology, Institute of Health and Biomedical Innovation, 60 Musk Avenue, Kelvin Grove, QLD 4059, Australia

E-mail: laura.bray@qut.edu.au

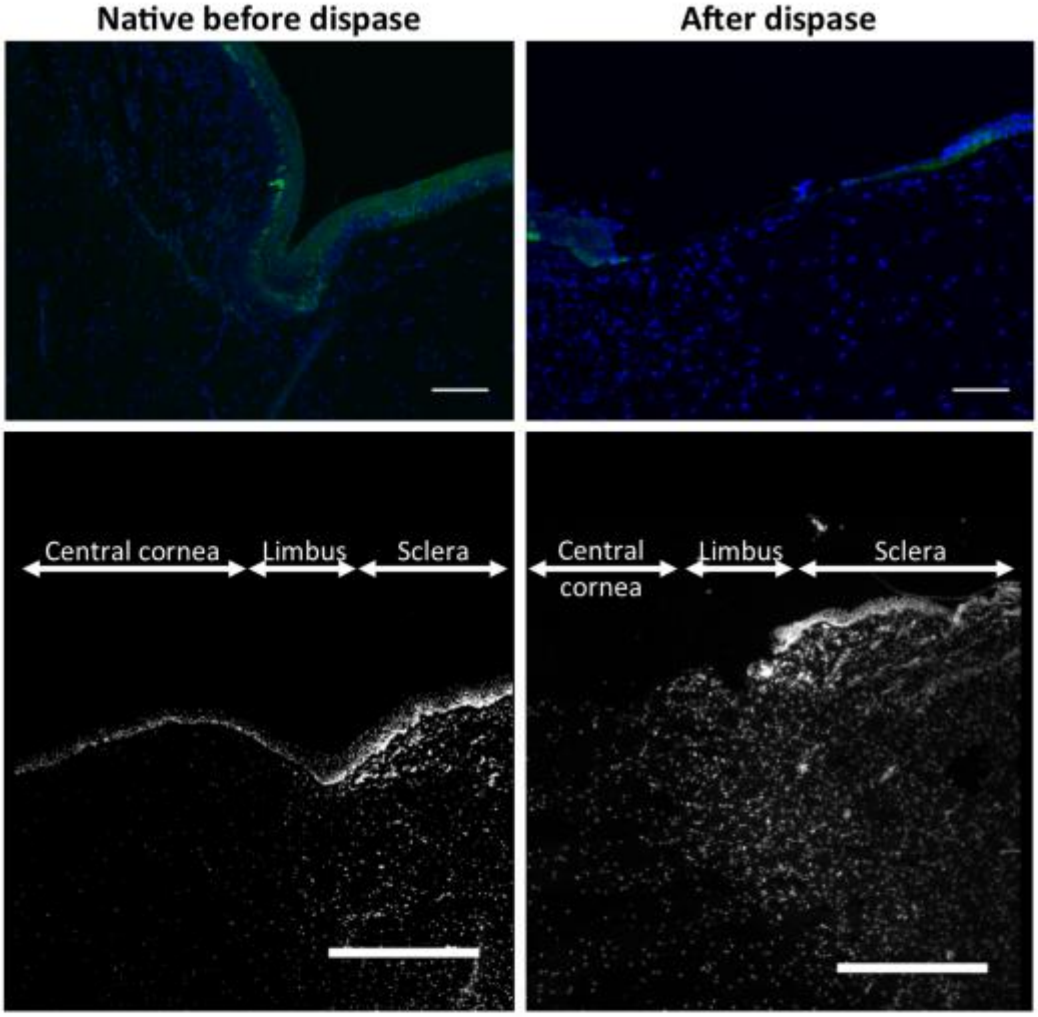

Supplementary Figure 1. Presence of pLESCs after treatment with dispase. Histological section of porcine cornea post-dispase. Green staining indicates ΔNp63 expression, blue staining indicates nuclei (top images). Scale bar = 100 µm. Bottom images display cornea section post-dispase and scraping. Staining indicates nuclei. Scale bar = 500 µm.

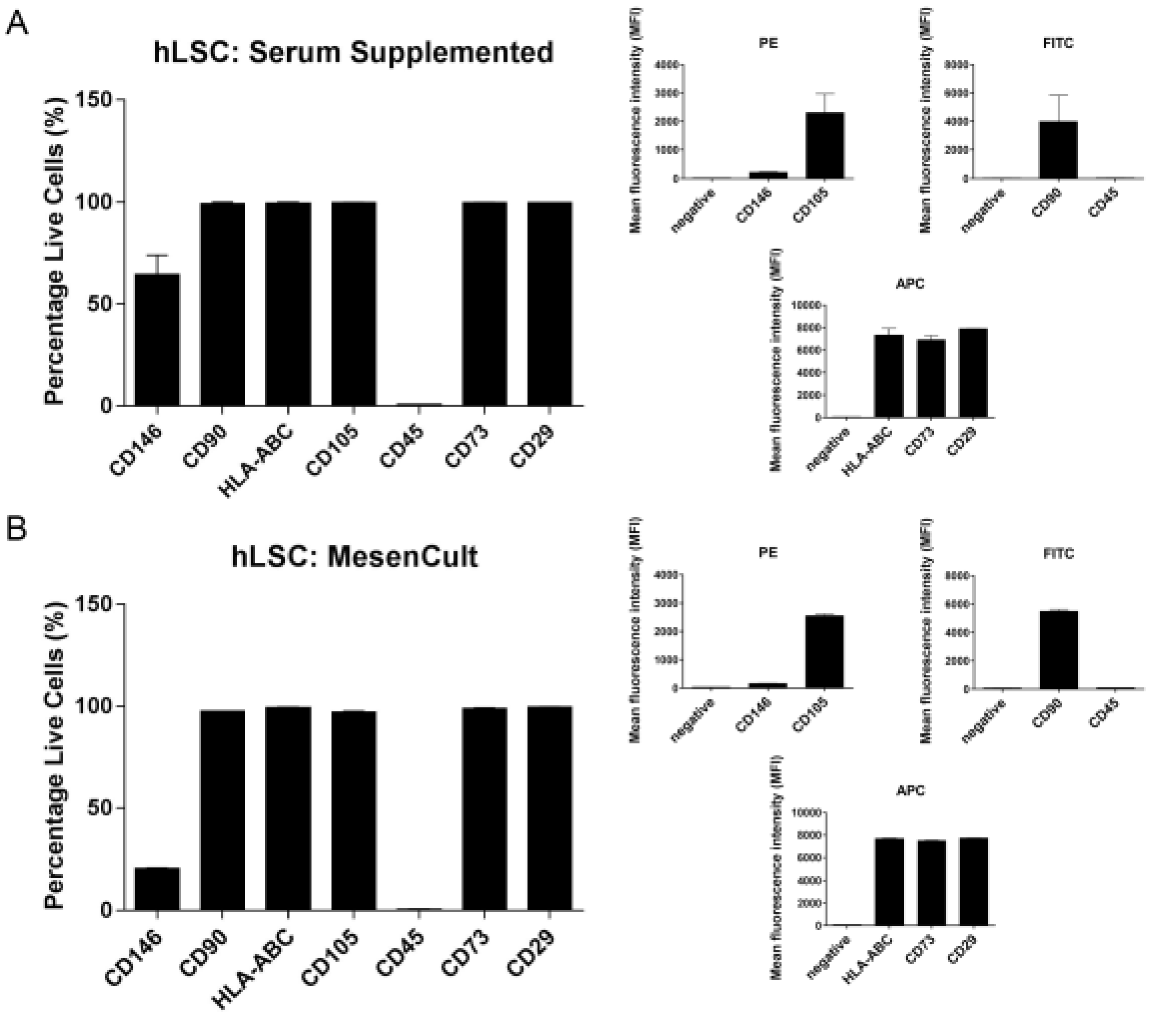

Supplementary Figure 2. Phenotyping of human limbal stromal cells by flow cytometry. HLSCs grown in medium containing 10% FBS (A) and hLSCs grown in serum-free MesenCult^{TM}-XF medium (B). Mean fluorescence intensities for each fluorophore are depicted on the right-hand side graphs. Data shown as mean ± standard deviation. Values were pooled from two biological replicates for hLSCs grown in 10% FBS and one biological replicate for hLSC grown in MesenCult^{TM}-XF, all performed in technical duplicates.

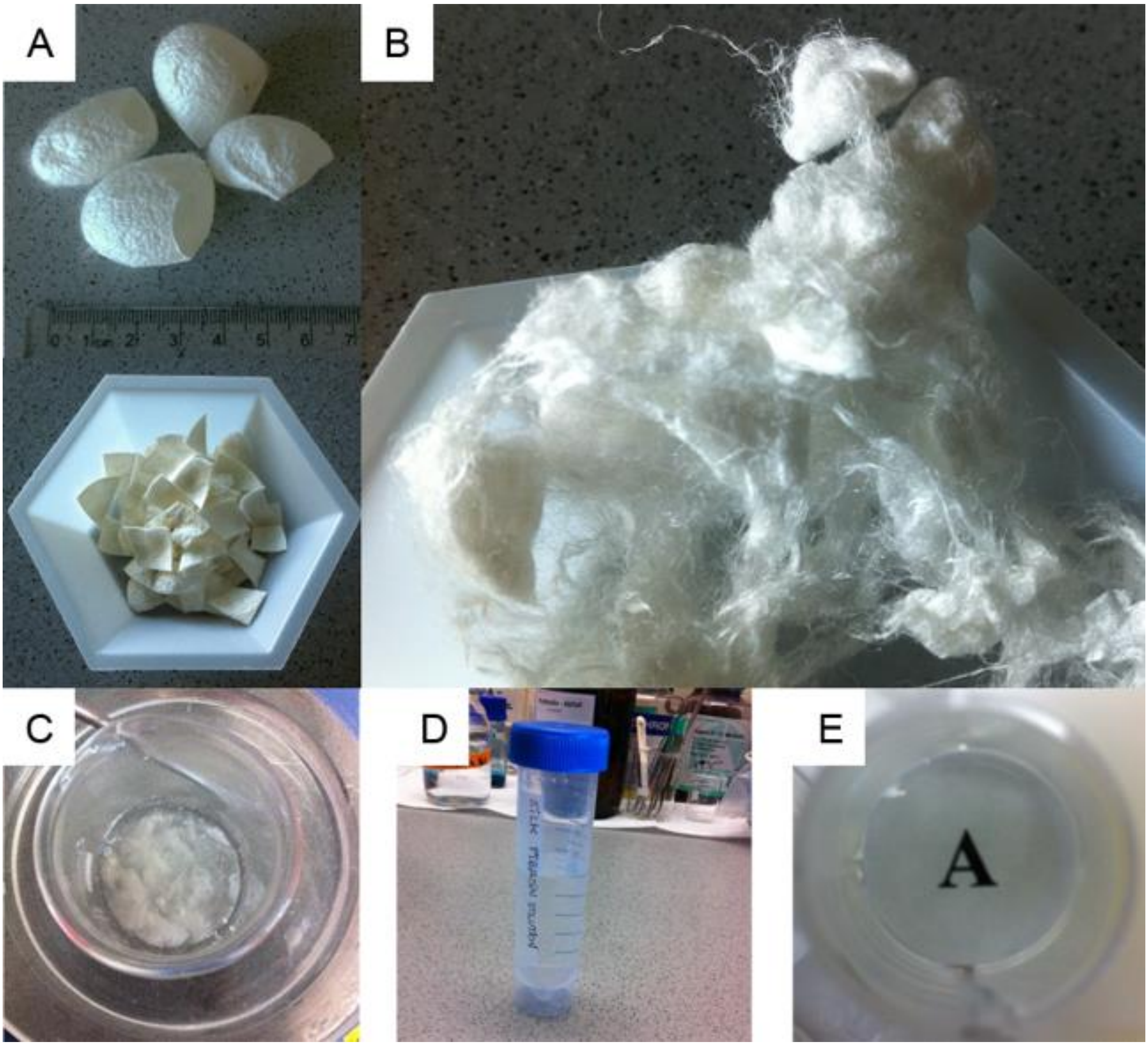

Supplementary Figure 3. Silk fibroin extraction. *Bombyx mori* cocoons (top) and cocoons cut into small pieces (bottom) (A). Degummed fibres (B). Solubilisation of fibres in LiBr solution (C). Silk fibroin solution (D). Silk fibroin membrane cast in culture well (positioned on printed text to show its transparency) (E).

Supplementary Table 1. Positive and negative expression of antigens on pLSCs cultured in either serum-supplemented medium or the MesenCult^{TM}-XF Culture Kit, as determined by flow cytometry. Positive expression indicates that the mean fluorescence intensity was above the negative control. All antibodies were anti-human with the exception of CD29, which was an anti-porcine antibody.

Antigen	pLSC: 10% FBS	pLSC: MesenCult	hBM-MSC: 10% FBS
**CD29**	+	+	-
**CD45**	-	-	-
**CD73**	-	-	+
**CD90**	+	+	+
**CD105**	+	+	+
**CD146**	+	+	+
**HLA-ABC**	+	+	+
